# Speech Treatment for People with Cerebellar Multiple System Atrophy (MSA-C): A Pilot Randomised Controlled Trial of Two Approaches

**DOI:** 10.1007/s12311-025-01895-y

**Published:** 2025-08-14

**Authors:** Anja Lowit, Kaiyue Xing, D. Priya Shanmugarajah, Emma Foster, Suzanna Duty, David Young, Jan Stanier, Christopher Kobylecki, Marios Hadjivassiliou

**Affiliations:** 1 University of Strathclyde, Glasgow, UK; 2 University of Sheffield, Sheffield, UK; 3 Sheffield Ataxia Centre, Sheffield, UK; 4 NHS Greater Glasgow and Clyde, Glasgow, UK; 5 Northern Care Alliance, Salford, UK; 6 University of Manchester, Manchester, UK; 7 Department of Psychological Sciences and Health, University of Strathclyde, Glasgow, G1 1QE UK

**Keywords:** Multiple system atrophy, Dysarthria, Ataxia, Rehabilitation, Speech therapy, Telehealth, Randomised controlled trial

## Abstract

Speech problems are an early feature of Multiple System Atrophy (MSA). They can lead to social withdrawal and have significant impact on people’s quality of life. There is a considerable lack of clinical trials and clinicians lack guidance on how best to support this population. This project aimed to establish the feasibility and acceptability of a novel treatment approach, ClearSpeechTogether, in patients with the cerebellar variant of MSA (MSA-C), and to pilot an RCT comparing this treatment to standard speech and language therapy (SLT) treatment (ST). We recruited 24 patients with clinically probable MSA-C and dysarthria who were randomised to either treatment arm. Full data were available for 9 participants for ST, and 11 for ClearSpeechTogether. Both interventions lasted 6 weeks, ST offered 1 h of individual therapy a week, ClearSpeechTogether provided four individual therapy sessions over two weeks, followed by four weeks of daily, patient led group practice. Assessment and intervention were provided online via videoconferencing software. Data collection focused on feasibility, acceptability and signal of efficacy. Recruitment, conversion and attrition rates were within or close to target, and neither participants nor clinicians highlighted any acceptability issues. Communication outcomes were mixed, with biggest gains made in communication confidence and participation across both groups. Rapid decline in overall health status appeared to have impacted results. Results were generally positive and support the implementation of larger follow up trials. The study also demonstrated that people with MSA-C can benefit from speech therapy even at more severe stages of their disease progression.

**Supplementary Information:**

The online version contains supplementary material available at 10.1007/s12311-025-01895-y.

## Introduction

### Background

Multiple system atrophy (MSA) is a neurodegenerative condition that presents in two predominant ways: Cerebellar variant (MSA-C) and Parkinsonian variant of MSA (MSA-P). Both phenotypes are associated with significant disability resulting in reduced lifespan. The Sheffield Ataxia Centre is one of a few clinics in the UK that specialises in the management of people with MSA-C. Data on over 100 patients with MSA-C collected at the Centre show that the mean age at symptom onset is 57 and the mean age of death 65 years. The disease often follows a rapidly progressive course, with median survival rates generally reported between 7 and 10 years [1, 2]. As such symptoms tend to appear more rapidly than observed in other more slowly progressing conditions [3, 4]. Patients often end up requiring use of mobility aids such as wheelchairs and in addition to the progressive ataxia, typically develop autonomic dysfunction features such as urinary urgency and frequency, postural hypotension and sleep problems such as sleep apnoea and REM-sleep disorder.

One of the early and distinctive symptoms of both types of MSA are speech problems such as dysarthria [3]. Irrespective of subtype, speakers tend to experience speech problems associated with hypokinesia, spasticity and ataxia. MSA-P is associated with hypokinetic symptoms such as harsh, quiet voice and articulation difficulties, MSA-C is more likely to present with motor coordination and control issues [5–7]. Spasticity manifests as slow and effortful speech production in both phenotypes [5, 6], and patients sometimes present with stridor [8, 9]. These speech problems result in difficulties communicating their daily needs, which in turn can lead to social withdrawal, impacting on people’s quality of life and mental health [10, 11].

Due to the early onset and relative severity of their dysarthria [6, 8], people with MSA should be a priority for speech and language therapy (SLT) input. However, until recently, no evidence was available about effectiveness of speech intervention for this patient group. There is now a growing body of small clinical trials demonstrating the potential benefits of intervention, mostly for MSA-C [12–15], although a recent single case study also reports positive effects of Lee Silverman Voice Treatment (LSVT Loud) on a person with MSA-P [16]. These have demonstrated benefits for breath support [12, 14, 15], voice quality [13, 14], oral diadochokinesis [15], loudness and pitch range [13], intelligibility [13, 14] and vocal handicap [13]. Whilst valuable, the studies suffer from a number of limitations. First, outcome measures have not addressed all levels of the WHO International Classification of Functioning, Disability and Health (ICF) model, particularly with regard to the participation level. Second, no acceptability data have been reported to date despite treatment delivery being highly intensive. Third, no studies have compared different types of interventions or built in no-treatment phases to control their outcomes. The last two points are particularly crucial with regard to the MSA population. A recent large scale trial with people with Parkinson’s (PDCOMM [17]) reports that only delivery of an intensive programme (LSVT Loud^®^ [18]) resulted in meaningful improvement of vocal handicap, with standard NHS provision showing no better results than no treatment. This suggests that intensity plays a crucial factor in intervention outcomes, however, this has to be balanced with clients’ ability to engage in such programmes in view of their overall physical wellbeing. It is therefore important to establish to what degree intensive delivery is also key to successful communication outcomes in people with MSA and how acceptable it is.

The PDCOMM trial [17] also emphasises the need for further research on other ways of delivering intensive treatment to reduce health service costs and the pressures this puts on service providers. Our research group recently developed a novel treatment model, ClearSpeechTogether [19] which could provide a viable alternative. ClearSpeechTogether is an interactive, patient centric telehealth model of care that combines individual SLT intervention with peer supported group therapy. The individual sessions establish relevant speech strategies whilst the group sessions empower participants towards self-management by providing opportunities for regular speech practice, development of self-monitoring skills, and internalisation of strategies and carry over into everyday communications. Furthermore, they provide a platform for social support and help to build confidence. The model is cost-effective, providing intensive delivery of 24 client sessions over six weeks with the input of only five to six clinician sessions and as such aligns well with current drivers to make healthcare more sustainable and less resource intensive, such as the principles of realistic medicine and values based healthcare [20, 21] as well as the conclusions of the PDCOMM trial [17]. Telehealth delivery minimises the impact of the scheduling intensity during the group phase. The results of the pilot study with people with progressive ataxia were positive, particularly for intelligibility and communication confidence and participation [19].

The purpose of the current study was to establish whether similar levels of communication benefit could be achieved in people with MSA-C. Furthermore, we wanted to assess the acceptability of the approach in this group, and whether it was feasible to conduct a two arm RCT evaluating the potential benefits of ClearSpeechTogether compared to the intervention patients with dysarthria are typically offered by UK healthcare providers (standard SLT (ST)) [22]. The trial thus investigated three aspects:

1. What is the feasibility of performing a larger scale RCT of ClearSpeechTogether compared to ST in a population of people with MSA-C?
2. What is the acceptability of the approaches to both participants and healthcare providers?
3. What are the potential communication and psycho-social benefits of the two approaches for people with MSA-C?

## Method

### Trial Design

This study consisted of a single, rater blinded, mixed method parallel group pilot RCT (Trial registration: ISRCTN44652664, registration date 16/12/2022). Participants were allocated to two intervention arms in a 1:1 ratio. Arm 1 consists of ClearSpeechTogether, Arm 2 acted as the control arm providing six sessions of individual SLT simulating the current provisions in the UK national health service, the NHS [22]. Four assessments were conducted, two prior to intervention, one immediately post-intervention and a 2 months follow up. No changes were made to the original protocol [23] with exception of some data evaluation methods as discussed below.

### Participants

Participants had a diagnosis of clinically probable MSA-C using established criteria [24]. Inclusion criteria were the presence of mild to mild-moderate speech impairment (scores between 2 and 4 on the Scale for the Assessment and Rating of Ataxia (SARA [25]), speech scale). Other criteria were sufficient (corrected) visual and auditory skills to complete the assessment and therapeutic exercises, and ability or support to use video-conferencing software. Exclusion criteria included the presence of other health conditions that can affect communication (e.g. stroke), or a history of communication impairment (e.g. stammer). Those with cognitive impairment due to other conditions were also excluded.

The inclusion and exclusion criteria were established based on medical records and clinical assessment during the recruitment process.

Participants were identified by consultant neurologists and specialist ataxia nurses. Potential participants were approached either directly when visiting their clinic, or by sending out a study invitation letter. In some cases, this was followed up by a telephone call.

The majority of participants were sourced through the Sheffield Ataxia Centre. Training was provided to recruiters at the clinic to judge severity reliably. Consent was taken by the nurses in the Sheffield Ataxia Centre, or by the Strathclyde University research team for participants recruited through the remaining channels.

All interventions and assessments were conducted via videoconferencing in the participants’ homes.

### Interventions

Two types of interventions were provided – ClearSpeechTogether [23] (arm 1) and standard SLT (ST, arm 2). A team of two SLTs specialised in managing adults with neurogenic communication problems provided the treatment.

#### Arm 1 – ClearSpeechTogether

ClearSpeechTogether is a six week intervention programme that involves four sessions of individual intervention spread over two weeks, followed by four weeks of daily group sessions, resulting in 24 scheduled treatment sessions per participant. All sessions last around 60 min. During the group phase, the SLT is available for questions and has the option to offer additional sessions to individuals if necessary. Non-clinical support staff are available during the group sessions in case technical help is required to join the online sessions.

ClearSpeechTogether focuses on two evidence based speech strategies – LOUD and CLEAR [26, 27]. Detailed information on the structure and contents of the programme are available from the programme resources [28, 29]).

#### Arm 2 – standard SLT

Arm 2 comprised of six individual 60 min long sessions delivered once a week. The focus of these sessions was decided by the SLT, with the guidance that the ultimate goal of treatment was to improve intelligibility and communication participation and for participants to be working at paragraph reading / free speech level by the end of the treatment if possible. A review of the clinical notes indicates that treatment strategies in arm 2 were largely comparable to arm 1 (LOUD and CLEAR), but additional concepts were introduced in some cases to achieve these strategies, such as focusing on speech rate reduction or stress production. ST also provided more opportunity to provide individually tailored exercises such as script training for situations the participants found difficult, e.g. making phone calls.

### Outcomes

The main goal of our study was to evaluate whether a future trial investigating the superiority of ClearSpeechTogether is warranted, both in relation to potential communication benefits and feasibility and acceptability for people with MSA-C and healthcare providers. We employed a mixed method design collecting qualitative as well as quantitative data. Measures included the following:

#### Feasibility

- conversion to consent (considering patient consent and fit with inclusion criteria, target = 75% of those identified agree to participate);
- rate of recruitment (number of consenting participants in 6 months, target = 24);
- rate of attrition (target = 75% retention rate);
- data quality (target = 75% of participants’ own recordings and 90% of researcher back-up recordings of sufficient quality for analysis);
- access to telehealth (target = 75% of those consenting have access to necessary technology and support to use it).

#### Acceptability

Participants

- adherence to the therapy programme (target = 80% attendance);
- fidelity to treatment programme (home practice diary - target = 75% completion of daily exercises (assuming some over-reporting); observation of engagement during peer group sessions);
- fatigue levels (target = less than 10% decline in overall fatigue level on the Fatigue Impact Scale attributed to participation);
- qualitative feedback regarding the appropriateness of the exercises, balance between individual and group sessions (Arm 1), quality of support provided in sessions, and the scheduling intensity of the sessions.

Clinicians

- fidelity to treatment programme: evaluation of 20% of session recordings;
- need for additional individual or group support (target = no more than 1 additional session required per participant);
- qualitative interview feedback regarding workload management;

#### Potential for Efficacy

Communication benefits were assessed across all ICF levels, including physiological (breath support and voice quality in sustained phonation and connected speech), functional (intelligibility in reading and free speech tasks) and participatory levels (communication confidence (1–10 scale) and participation (Communication Participation Item Bank, CPIB [30]).

Communication confidence and participation were the primary outcomes. Intelligibility, maximum phonation time (MPT) and voice quality were secondary outcomes. Some changes had to be made to the evaluation methods for these measures as reported in the study protocol [23]. Our original targets were a 20% increase in CPIB [30] and confidence scores to indicate that a full RCT is warranted. However, it became apparent during data analysis that percentage scores were meaningless and the absolute point difference achieved by each individual was used instead. The CPIB contains 10 items, improving by one point across at least half of these would result in an increase of 5 points, which we selected as our threshold for clinically relevant change. For confidence we looked for an improvement of 2 points. We validated these by comparing participation and confidence scores with qualitative data from participant reports on noticeable change from the ClearSpeechTogether pilot study [19]. The figures also broadly correspond to change thresholds reported for other patient reported outcome measures such as the Voice Handicap Index [17, 31]. Further changes to outcome measures were introduced for intelligibility and voice quality. Original plans had been to analyse intelligibility by means of direct magnitude estimation [32] and voice quality with the CAPE-V [33] which uses a visual analogue scale (VAS) for scoring. This decision was based on a pilot exercise with a group of ten trained listeners evaluating 12 samples from the participant pool representing a range of speech impairment levels using a variety of evaluation methods including published Likert scales (ranging from 4 to 10 points), percentage scores, VAS and DME. Of these, DME resulted in the highest inter-rater reliability scores with an ICC of 0.977. In addition, the CAPE-V has established inter- and intra-rater reliability data for the VAS scoring method and is reported to be more sensitive than the scalar GRBAS equivalent [34, 35]. However, despite running several consensus exercises with the expert listeners for the final project evaluation, we were unable to achieve satisfactory levels of inter-rater reliability using these measures (DME ICC: 0.368, CAPE-V ICC: 0.431, values below 0.5 are considered poor), potentially because we were using less evaluators at that stage. We therefore adopted scalar methods instead, using the SARA speech score for intelligibility, which had also achieved high reliability results in our pilot study (ICC = 0.948), and the GRBAS score for voice quality. This resulted in medium to high reliability outcomes for the ICC for both inter- and intra-rater reliability (reading intelligibility: inter-rater ICC = 0.773, intra-rater ICC range: 0.642 − 0.873, GRBAS: inter-rater ICC = 0.672, intra-rater ICC range: 0.448 − 0.815). We defined a 0.5 change in scores as being clinically relevant, which meant that at least 50% of our listeners had perceived a change of at least one point on the scale. MPT was measured in seconds, and we defined a 20% change in scores as being clinically relevant.

### Sample Size

As this was a pilot trial, a sample size calculation was not necessary. A target of 24 participants was determined pragmatically by (1) the plan to run two groups of ClearSpeechTogether with six participants in each, i.e. 12 participants in total, with matched numbers in the ST arm, and (2) the limited number of potential participants fulfilling the selection criteria, given the rare nature of the condition.

### Randomisation

Following recruitment, participants were randomised by an independent statistician in a 1:1 ratio to both arms. Arm 2 participants were offered to join a speech support group at the end of the trial if they wished.

An independent statistician generated the allocation sequence using block randomisation with block size 4, using Sealed Envelope (www.sealedenvelope.com). Recruiters were based in a different centre and had no knowledge of the allocation sequence.

### Blinding

Given the different nature of the interventions provided it was not possible to blind participants, SLTs or interviewers to the type of therapy they were allocated to. However, all speech assessors and data analysists were blinded to the timing of assessment and type of intervention.

### Data Collection, Management, and Analysis

All data collection sessions were conducted using video-conferencing software, including Zoom, and occasionally WhatsApp and Facebook messenger.

Audio and video recordings of the assessment were made using the native recording facility of the Zoom application. To achieve better recording quality for the speech assessment, participants also recorded these tasks with a custom web-application that saved recordings directly to a secure Microsoft OneDrive account. Where this was not possible, the zoom audio was used for analysis.

Quantitative data were analysed with non-parametric statistical tests, using the Friedman test to detect changes over time within each treatment arm, and Wilcoxon Signed Rank tests to conduct post-hoc analyses where relevant. Group differences were assessed with the Mann-Whitney-U-test. In addition, due to the relatively small group sizes, effect sizes were calculated to supplement the p-values. Finally, we supplemented the statistical analysis by descriptively assessing how many speakers in each group showed positive changes beyond the clinically relevant threshold. For this purpose, we compared each of the post-treatment assessment scores with their time matched pre-therapy assessment (PT) in order to exclude time of day impacting on the performance analysis.

With regard to the qualitative feedback, an extensive thematic analysis had been carried out for the previous pilot study of ClearSpeechTogether. The same interview guide was used for the current study. Therefore, instead of performing an independent analysis, we cross-checked current feedback against the established themes, focusing on additional information provided or diverging views.

### Ethics

The study was conducted in accordance with the Declaration of Helsinki and was awarded ethical approval by the UK National Research Ethics Service, IRAS project ID 322,064. All participants provided informed consent.

## Results

### Participants and Recruitment

**Fig. 1:**
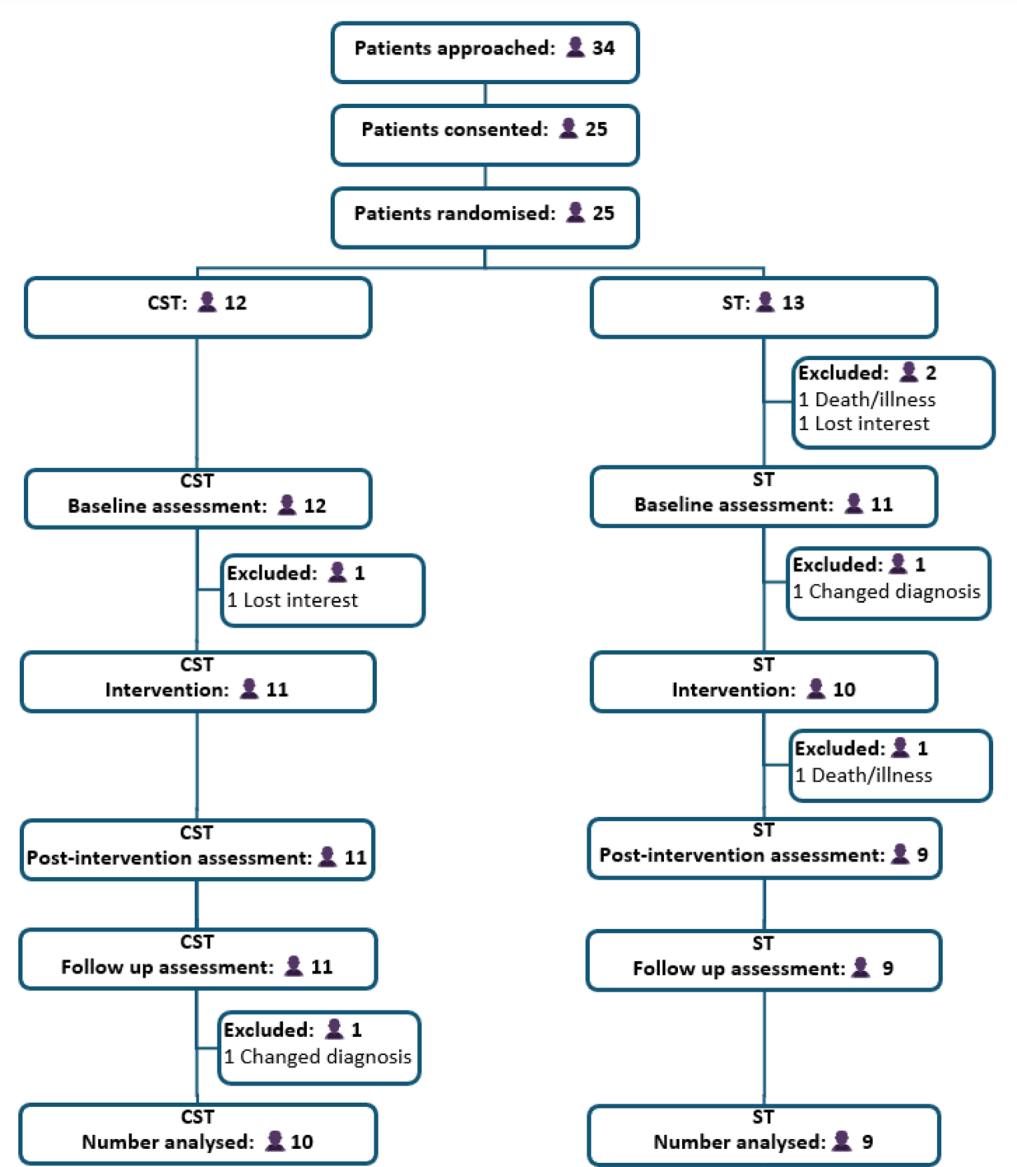
Trial flowchart. Abbreviations: CST: ClearSpeechTogether, ST: standard SLT

The trial flowchart in Fig. 1 summarises the conversion and attrition data for the study. Thirty four people with probable MSA-C were approached over a 10 month period from April 2023 to February 2024 through three NHS sites and the MSA charity. Twenty five of these consented to participate and were randomised to the two intervention arms − 12 to ClearSpeechTogether and 13 to ST. This translates to a conversion rate of 74%, thus narrowly missing the target of 75%. Those who declined to participate and provided a reason generally indicated that were worried about not being able to participate in the intensive ClearSpeechTogether arm of the study. The recruitment period exceeded the planned 6 months due to staffing issues and the need to add additional patient identification sites to the project. Post-treatment data are available for 20 participants and 2 months follow up data for 19. Attrition occurred at various stages, due to death, illness, loss of interest and change in diagnosis from MSA-C to other conditions subsequent to recruitment. One participant in the latter category completed all stages of the trial and will be discussed separately below. The total attrition at follow up was 24%, which is within the target of less than 25%.

Table 1 provides demographics for the participants that were included in the study. The two groups were well matched in terms of baseline ataxia severity (SARA score, *p* = .806) as well as severity of their speech difficulties (SARA speech score, *p* = .233). Tables S1a&b in the supplemental materials provide further information on individual participants’ speech features, symptom onset and previous speech therapy input. All participants presented with dysarthria features in line with previous reports on MSA-C and most reported onset of speech difficulties early on in their disease process [5, 6]. Ataxic dysarthria symptoms such as irregular speech rhythm and articulatory breakdown prevailed, but some signs of hypokinetic dysarthria (fast rate, short rushes of speech, hypophonia (e.g. P1 & 3)) and spasticity (strained voice quality, stridor (e.g. P2)) were also evident across the samples.

**Table 1:** Participant demographics

ID	Arm	Gender	Age	Years since diagnosis	Ataxia severity (SARA)	Speech severity (SARA speech)	Deterioration reported
1	ST	female	67	1	26.5	2.3	?
2	ST	female	58	1	16.5	2.5	y
3	CST	female	75	2	21	3.8	y
4	CST	male	49	3	14.5	3.0	y
5	ST	female	59	4	18.5	2.3	y
7	CST	male	58	3	26	3.0	y
8	CST	male	57	1	13	2.5	y
9	CST	female	62	2	17	1.0	y
10	ST	female	61	5	20	3.8	N
11	ST	female	70	2	17.5	2.3	N
12	CST	female	67	3	16	2.5	y
13	ST	female	64	0	8.5	0.0	y
14	ST	male	79	0	13.5	0.5	y
15	CST	female	73	2	25.5	3.0	y
16	CST	male	67	0	24	3.8	N
17	CST	female	64	0	10	1.3	y
19	ST	female	69	0	16	1.8	y
21	CST	female	65	0	13	3.3	y
24	ST	male	69	3	25	4.8	?
CST	Mean (SD)	6 F, 4 M	63.7 (7.7)	1.6 (1.3)	18 (5.7)	2.7 (0.9)	Y: 9 N: 1
ST	Mean (SD)	7 F, 2 M	66.2 (6.6)	1.8 (1.8)	18 (5.5)	2.26 (1.5)	Y: 5 N: 2 ? 2

Abbreviations: ID: participant identifier, CST: ClearSpeechTogether, ST: standard SLT, F: female, M: male, SD: standard deviation, SARA: Scale for the Assessment and Rating of Ataxia, Y yes, N: no, ?: no information available

### Feasibility

As stated above, conversion and attrition were at or close to target. However, we had only recruited 16/24 participants at the end of the 6-month recruitment period. At the time, we had exhausted the existing caseload of MSA-C patients registered with the Sheffield Ataxia Centre and depended on incoming newly diagnosed patients as well as adding other sites to the recruitment pool, extending the recruitment period to 10 months in total.

In relation to data quality, we experienced no data loss or quality issues. However, collecting speech recordings online was difficult for some participants and added additional stress. In future trials it would be preferable to have in person assessments for speech recordings.

Digital exclusion was not a concern in the current cohort, all participants were able to join the online sessions, less than 10% required provision of the necessary hardware and all were able to operate the tools with guidance. There was no evidence that the online treatment provision had adverse effects on participation.

The maximum wait time for ClearSpeechTogether participants was around 4 months and no participants dropped out of the study for this reason.

### Acceptability

#### Participants

The target was to achieve a minimum 80% attendance rate for treatment sessions. In the ST group, attendance was 100% as sessions were rescheduled in case of issues. ClearSpeechTogether participants attended all individual sessions and at least 4 of the 5 group sessions per week. Two participants missed an entire week of group sessions due to illness or other commitments, lowering the overall attendance to 84% in this arm.

Only a small subset of homework diaries were returned, but those who did showed the expected level of homework practice. Those who did not complete the diary were asked verbally during the post-treatment interview and again indicated that they had been able to do at least some self-practice in between sessions. Daily observations of the participant led group sessions by the researcher indicated that all participants contributed equally to the group across the four weeks and performed the exercises as intended.

Fatigue levels were collected pre- and post-treatment largely to monitor for any adverse effects of the intensive group phase of ClearSpeechTogether. Whilst some participants reported increased fatigue levels post-treatment, this was not attributed to the effects of the therapy, but the overall worsening of their condition. Overall, Fatigue Impact Scale scores were relatively stable across both arms, with most participants reporting no change (ST: 78%, CST: 50%). A small number in each group reported feeling more tired afterwards (ST: 11%, CST: 20%), but there were also some who reported an improvement in fatigue levels (ST: 11%, CST: 30%). There is therefore no evidence that the intensity of the ClearSpeechTogether treatment had an adverse effect on participant wellbeing.

Qualitative feedback on the structure and scheduling of the treatment was largely positive for both arms. All participants felt that the treatment was appropriate to address their concerns about their speech and met their needs. Duration of the treatment was appropriate. Two of the eleven ClearSpeechTogether participants indicated that two sessions per week would have been sufficient during the group phase. Despite these reservations, their attendance was high and no adverse effects were noted. Many ClearSpeechTogether participants felt that the intensity had been important to the effectiveness of the treatment. Nobody reported any issues with working with the other members of the group and the observer indicated good dynamics and a supportive atmosphere throughout the sessions. Some participants felt that face to face treatment would have been preferable, but that this was outweighed by the fact that they did not have to travel to clinic for their session.

Finally, an important aspect of ClearSpeechTogether is that it is intended to provide longer term support by individuals continuing to meet after the intervention concludes. Of the two groups, the first met a further three times but participants then did not attend further, mostly for health reasons. The second group continues to meet on a monthly basis 12 months on from completing the intervention.

#### Clinicians

Two SLTs were involved in the treatment, but one only saw two ClearSpeechTogether participants for their four individual sessions. The interview was therefore only conducted with one SLT who reported that both treatment arms worked equally well in relation to being able to address patient need. As indicated, the strategies advised to participants across the two arms was relatively similar, however, ST allowed the inclusion of more tailored tasks to address participant’s areas of difficulty. The number of sessions provided were sufficient in both arms to reach the set goals, and no additional sessions had to be organised for ClearSpeechTogether participants during the group phase. ClearSpeechTogether was seen as valuable in providing group support and there were no concerns over adverse effects introduced by the participant led sessions. ClearSpeechTogether was considered superior in terms of clinician workload in relation to patient input and intensity, however, administration time to organise and support the group sessions would need to be considered in the health economic evaluation were the programme to be offered through the health service in future.

### Signal for Efficacy

#### Primary Outcomes

Communication confidence and participation were the primary outcome measures. There were no significant differences at baseline for either measure (confidence: *p* = .080, CPIB: *p* = .870).

Both measures showed significant differences over time for both study arms (Table 2). Post-hoc analyses demonstrate that these changes mostly occurred between A1 and A3. Differences between A3 and A4 were not significant, indicating maintenance over time.

**Table 2:** Means and standard deviations and statistical results for pre- and post-therapy assessments for confidence and participation scores

Summary Data		CST			ST			
**Assessment**		A1	A3	A4	A1		A3	A4
Confidence	mean	2.60	5.30	5.00	5.22		6.33	6.38
	SD	1.58	2.11	3.08	3.23		2.74	2.26
Participation	mean	8.20	12.85	12.72	8.78		12.72	13.75
	SD	7.10	4.38	6.95	7.64		7.05	6.11
**Statistical Results**	**CST**				**ST**			
	Friedman	A1-A3	A1-A4	A3-A4	Friedman	A1-A3	A1-A4	A3-A4
Confidence	**0.008**	**0.013**	0.068	0.798	**0.031**	**0.031**	0.410	0.581
Participation	**0.022**	**0.024**	0.058	0.859	**0.015**	**0.035**	**0.008**	1.000

Abbreviations: CST: ClearSpeechTogether, ST: standard SLT, A: assessment, SD: standard deviation. Statistically significant values are marked in bold

**Fig. 2:**
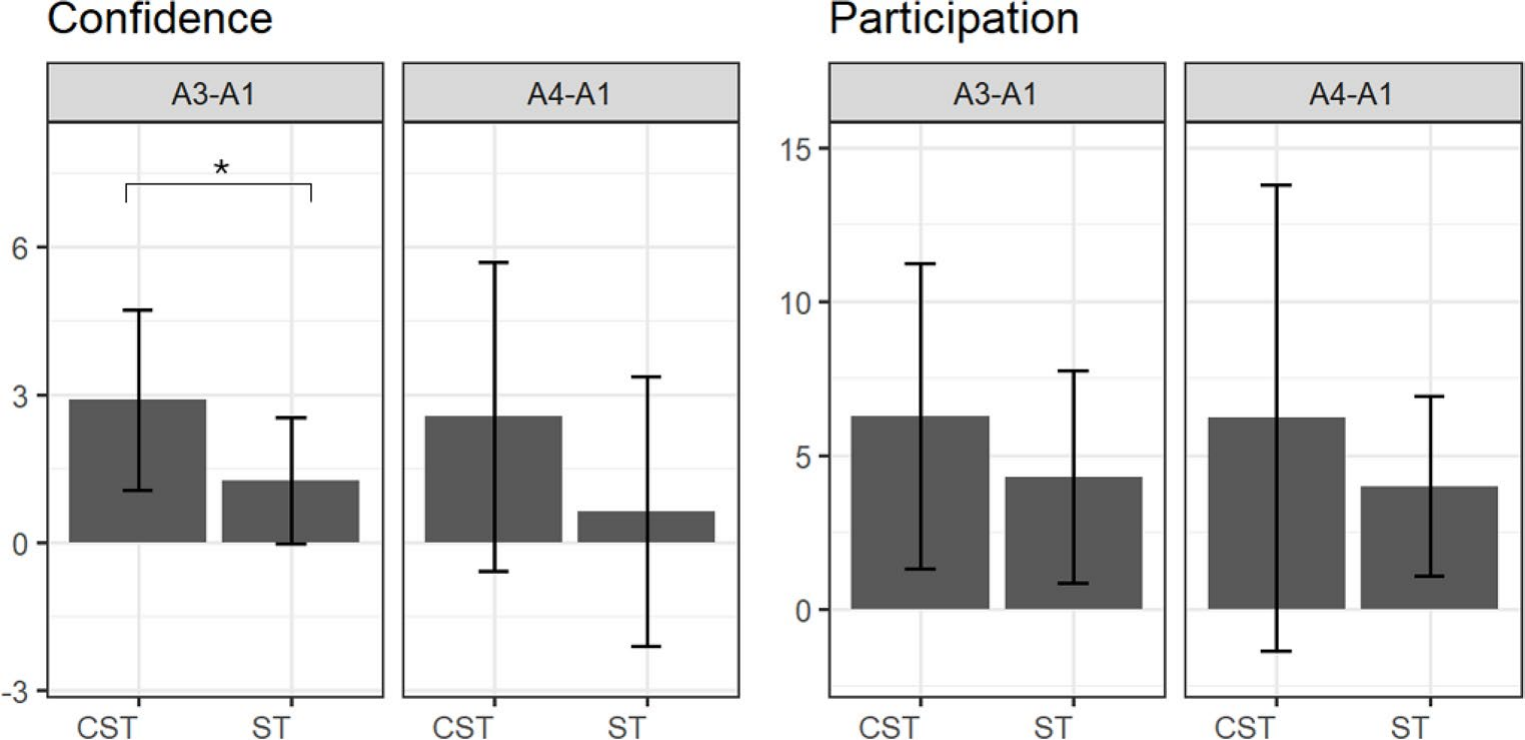
Comparison of the absolute magnitude of change between pre- and post-therapy scores for ClearSpeechTogether and standard SLT. Abbreviations: CST: ClearSpeechTogether, ST: standard SLT, A: assessment

Group comparisons of the degree of change between pre- and post-intervention showed that the ClearSpeechTogether arm made significantly greater improvements than the ST participants for confidence between A1 and A3, and the effect size calculations (d) suggests a further potential difference between A1-A4 with a medium effect size. The remaining comparisons were not significant (confidence: A1-A3 *p* = .034, d = 1.016; A1-A4 *p* = .119, d = 0.682; CPIB: A1-A3 *p* = .414, d = 0.144; A1-A4 *p* = .563, d = 0.384, Fig. 2).

**Fig. 3:**
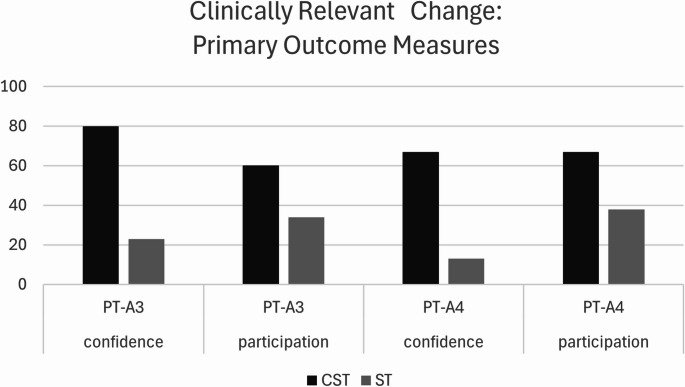
Percentage of participants who improved beyond the clinically relevant change thresholds for confidence and participation between pre-therapy (PT) and immediate (A3) and follow up post-therapy assessments (A4). Abbreviations: CST: ClearSpeechTogether, ST: standard SLT, PT: pre-therapy, A: assessment

Further group differences emerged from the descriptive analysis considering clinically relevant thresholds for change in that a higher proportion of ClearSpeechTogether participants achieved an increase at or above the threshold (Fig. 3) for all comparisons.

The data thus suggest that both treatment arms successfully improved communication confidence and participation in many participants, and that these effects were generally maintained at follow-up. Both statistical and descriptive analyses suggested superiority of ClearSpeechTogether over ST in this respect.

#### Secondary Outcomes

Intelligibility, breath support and voice quality were secondary outcome measures.

Intelligibility was evaluated in both reading and free speech tasks. The two arms were well matched at baseline (reading: *p* = .269, free speech: *p* = .389). There were no significant differences over time for either group or task (Table 3). The ST group showed a worsening of performance in reading at A4 which approached significance.

**Table 3:** Means and standard deviations and statistical results for Pre- and Post-Therapy assessments for reading and free speech intelligibility scores and maximum phonation time (MPT)

Summary Data	CST						ST				
**Assessment**		A1	A2	A3	A4	F	A1	A2	A3	A4	F
MPT	mean	6.87	5.39	9.95	6.68	0.172	8.27	6.36	6.89	6.84	0.861
	SD	4.49	2.45	5.87	4.60		8.27	6.05	3.98	4.21	
Reading Intelligibility	mean	2.31	2.31	2.14	2.33	0.705	2.09	1.98	2.04	2.25	0.058
	SD	1.20	1.27	1.14	1.35		1.49	1.44	1.49	1.48	
Free speech Intelligibility	mean	2.70	2.48	2.60	2.39	0.547	2.48	2.52	2.52	2.81	0.098
	SD	1.57	1.52	1.65	1.56		1.63	1.72	1.55	1.80	

Abbreviations: CST: ClearSpeechTogether, ST: standard SLT, A: assessment, SD: standard deviation, F: Friedman test results

**Fig. 4:**
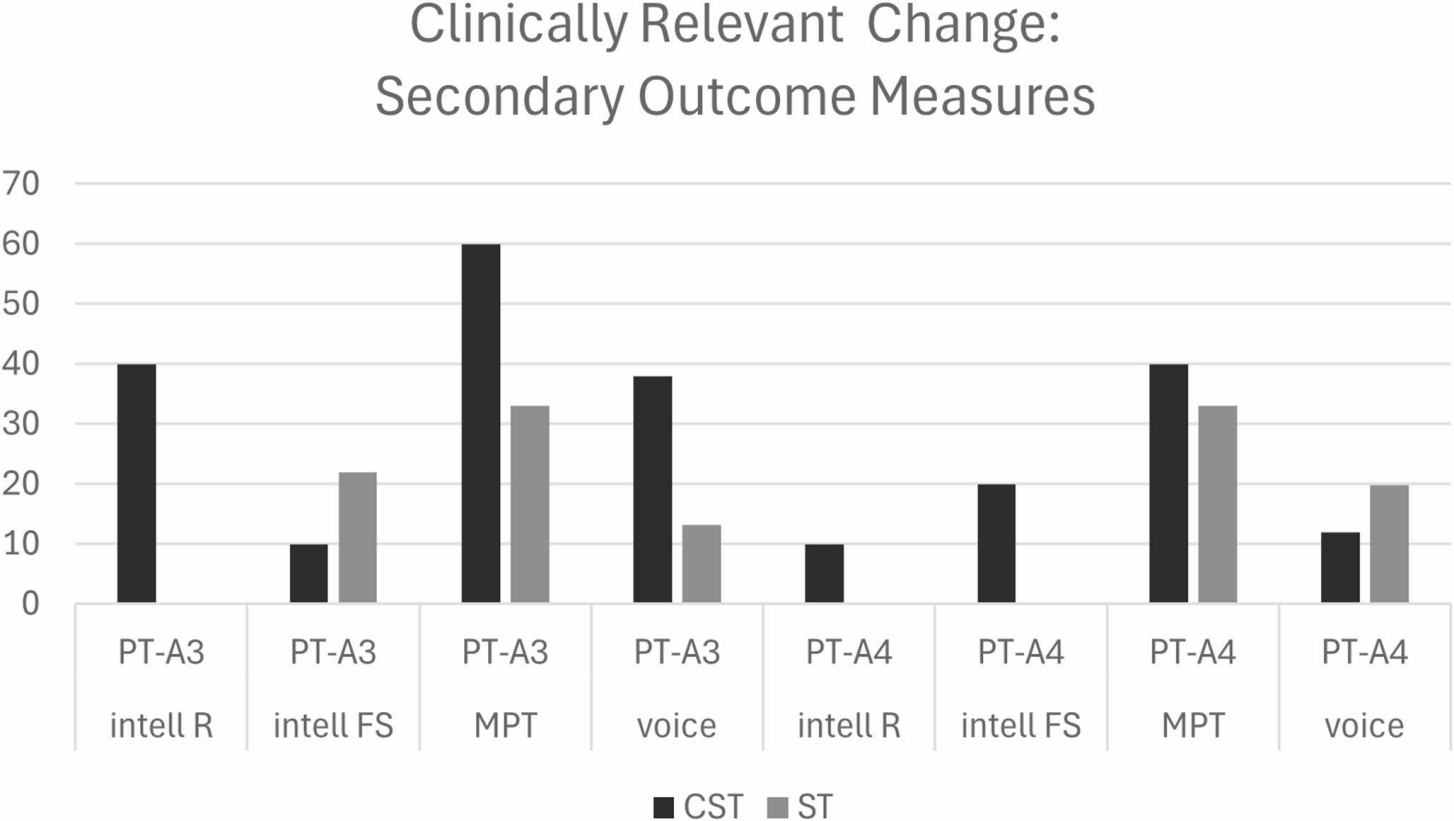
Percentage of participants who improved beyond the clinically relevant change thresholds for intelligibility, breath support (MPT) and voice quality measures between pre-therapy (PT) and immediate (A3) and follow up post-therapy assessments (A4). For voice quality, the figure presents the mean value across all five GRBAS scores. Abbreviations: CST: ClearSpeechTogether, ST: standard SLT, PT: pre-therapy, A: assessment, intel: intelligibility, R: reading FS: free speech, MPT: maximum phonation time

There was no difference in the degree of improvement from pre- to post-intervention between the two arms in either reading (PT- A3: *p* = .287, d = 0.742; PT-A4: *p* = .102, d = 0.169) or free speech (PT- A3: *p* = .743, d = 0.181; PT-A4: *p* = .062, d = 0.617). However, the effect size analysis (d) suggested a medium to large potential for ClearSpeechTogether to show greater improvement for the PT-A3 comparison in reading, and PT-A4 in free speech. The descriptive analysis further supports this, indicating that a higher number of ClearSpeechTogether participants improved above the clinically meaningful threshold in reading at A3 (Fig. 4).

Breath support was evaluated on the basis of an MPT task. Groups were again well matched at baseline (*p* = .870). There was no difference over time for either group (Table 3) and group comparisons showed no significant differences in the degree of change (A3: *p* = .094, d = 0.634; A4: *p* = .810, d = 0.331) although there was a medium effect size for ClearSpeechTogether participants showing greater improvements at A3. Twice as many ClearSpeechTogether than ST participants improved their MPT beyond the 20% threshold across the two groups at A3, but there was no difference by A4 (Fig. 4).

The final analysis parameter was voice quality in sustained vowels and connected speech (supplemental materials: Tables S2a&b). Baseline levels were again comparable between groups (*p* = .454). Analysis over time indicated no significant differences. Group comparisons and descriptive analysis showed a small advantage of ClearSpeechTogether over ST at A3 but not A4 (Fig. 4). The GRBAS categories demonstrating the highest level of improvement in the ClearSpeechTogether group were roughness and strain (50% of participants), no particular pattern emerged in the ST arm.

In summary, there were no statistically significant changes apparent over time for the secondary outcome measures. Effect sizes and descriptive analyses suggested potential for ClearSpeechTogether to have greater benefit for participants for intelligibility and breath support but this requires further investigation.

Despite the non-significant statistical results, qualitative reports on the secondary variables were generally positive (Supplemental materials: Table S3). Only two participants indicated no or negligible changes in their speech, the rest reported a range of benefits, with a stronger voice and better breath management featuring most frequently. There were no noticeable differences between the two study arms in this regard.

Given that our sample include a wide range of disease severities, it was possible that this factor might have influenced the therapy outcomes. We therefore correlated both motor and speech severity values with the degree of change from time-matched pre-therapy to immediately post-therapy (A3) outcomes. Whilst the total SARA and speech SARA scores were related to each other (*r* = .502, *p* = .028), we found no correlation between either of these severity scores and any of the primary or secondary outcome measures.

Finally, as indicated above, one ClearSpeechTogether participant had completed all stages of the trial before his diagnosis was changed from probable MSA-C to MSA-P and a full data set was therefore available for him. The results show that he also responded well to the intervention, his reading intelligibility improved beyond the threshold and this was maintained during the follow up assessment, and he was one of the few participants who showed improvements in monologue intelligibility. He also reported clinically relevant improvements in confidence and voice quality measures.

## Discussion

This study investigated the feasibility of conducting a randomised controlled trial comparing two types of speech therapy intervention as well as the acceptability and potential effectiveness of either intervention in patients with MSA-C.

Feasibility and acceptability results were positive and largely met our expectations. In terms of feasibility, conversion to consent narrowly missed the target, mostly due to poorer conversion numbers in one of the participant identifier sites, who only sent out letters to potential participants without personal follow up. All other sites had conversion numbers in excess of our target. The issue highlights the importance of personal interaction during the recruitment process instead of relying on participants to respond to invitations by mail. Rate of recruitment was good until the existing case load of suitable participants was exhausted. The issue was successfully resolved by including further sites to maintain the flow of recruitment, but this would need to be considered in future trials given the rarity of MSA in the UK, particularly the cerebellar phenotype.

Conversion of eligible participants was mostly impacted by worries of not being able to deal with the intensity of the ClearSpeechTogether treatment. The current data show that these worries were in fact unfounded and even participants with more severe MSA were able to participate in all sessions without detriment to their fatigue levels. Qualitative comments about the structure of the ClearSpeechTogether programme were positive and matched those from the original pilot trial [23]. We were thus able to demonstrate feasibility to run a larger RCT comparing the currently employed intervention approaches.

Both interventions succeeded in improving communication confidence and participation immediately post-therapy and longer term. This was in line with the previous ClearSpeechTogether pilot [23]. No other study to date has reported on these factors. Sonoda et al. [15] included evaluations of vocal handicap (VHI [36]), but only identified a trend towards improved scores in a mixed group of participants with MSA-C and cerebellar cortical atrophy (CCA).

Impact on the secondary outcome measures was not conclusive with participants’ performance both better or worse post-therapy, or, on the most part, not showing any changes, resulting in an absence of statistically significant changes. This could suggest that neither treatment was effective. On the other hand, most participants reported positive outcomes on their speech and the effect size analysis also suggested that larger samples size could have resulted in significant results in at least some parameters. Other studies have reported more definitive outcomes, e.g. our pilot of ClearSpeechTogether [23] found significant improvements in reading intelligibility, MPT and voice quality, Park [13] reports significant improvements of articulatory clarity and Chae et al. [14] report significantly improved intelligibility, MPT and voice quality. However, there are differences in our sample compared to these studies’ in terms of participant diagnosis or disease stage. For example, Park [13] studies participants with both MSA-C and MSA-P. Their therapeutic approach was originally developed for people with Parkinson’s and it is unclear from their reporting whether the positive improvements applied to both types of MSA or mainly those with MSA-P. Chae et al. [14] reported improvements only in participants with a disease duration of less than 4 years, suggesting milder severities may respond better to intervention. Whilst most of our participants were also diagnosed within 4 years, there was a considerable discrepancy between date of diagnosis and the onset of their symptoms which added an average of 4 years to their disease duration (Supplemental materials: Table 1a). It is thus possible that our group was more severely affected than Chae et al.’s [14]. Having said that, our data did not indicate a relationship between baseline motor or speech severity and intervention outcomes for intelligibility. In fact, three of the four participants who made clinically relevant gains presented with more advanced levels of baseline severity. This is an important factor to note in relation to clinical management to ensure the option of speech intervention remains open in addition to introducing alternative and augmentative communication aids in the later stages of disease progression.

A further consideration was that due to the rapid progression of MSA, many of our participants reported that their condition had declined within the six week period of their treatment (90% in the ClearSpeechTogether and 70% in the ST arm where this information was available, Table 1). In this context, it could be argued that a result of “no change” post-therapy can be considered a successful outcome as speech performance was maintained in the presence of overall decline. Qualitative feedback from participants supported this assumption, indicating that they felt their speech would have been worse had they not taken part in the programme. This issue highlights that close monitoring of health status is critical to allow accurate interpretation of intervention outcomes in populations with rapid disease progression.

In summary, the data for primary outcome measures indicates good potential for efficacy for both interventions. The results for secondary outcomes did not reach significance although the effect size analysis suggested that this might change with larger participant numbers.

The comparison between the two intervention approaches showed no clear indication of superiority in the statistical analysis with the exception of the confidence measure. This might change again with larger participant groups as suggested by the effect size analysis and descriptive evaluation of clinically relevant changes, with ClearSpeechTogether potentially resulting in greater benefits to participants. Such an outcome would be in line with the findings of the PDCOMM trial [17] which suggests that intensive delivery plays an important part in intervention outcomes. Importantly, our study has demonstrated that patients at even more advanced disease stages can partake in intensive intervention, at least when delivered online in their own homes.

Our study suffered from a number of limitations, the most impactful being the fact that we did not formally control for disease status based on the assumption that this would remain sufficiently stable with the 4 months study period to not affect outcomes. For future research it is advisable to conduct an array of different measures, both clinical and self-reported reflecting disease severity pre-as well as post-therapy to be able to factor in changes in overall health status in the interpretation of results when working with people with MSA or other fast progressing conditions.

The later addition of other participant identification sites also introduced variability in the recruitment approach, resulting in the inclusion of the participant whose diagnosis was later changed, and a further two participants whose severity levels were milder than originally planned.

Due to time restrictions we were only able to extend the follow up period to 2 months post-intervention in this trial. For a full RCT, a period of at least 6 months, and preferable longer should be assessed to determine the full long-term efficacy of the intervention. This could create issues if participants with MSA are included due to the speed of decline in overall health status observed in this trial. To address this issue, inclusion criteria could be restricted to newly diagnosed patients or at least those at milder severity stages. In addition, it will be important to properly monitor progression during the trial as suggested above to be able to factor this into the statistical analysis. Further research into how speech and voice change alongside the progression of other symptoms would also allow a more accurate evaluation of intervention outcomes.

Finally, an important part of our analysis focused on how many speakers achieved clinically relevant changes. One of the limitations of previous research is that this is not always considered. Statistically significant results for pre- to post-therapy comparisons could theoretically be achieved by small differences in values, as long as sufficient participants change scores in the same direction. However, these might not actually have any impact on their communication. Thresholds therefore need to be set on what constitutes a meaningful change, but information on what these should be is severely lacking. The current study based its thresholds on a combination of previous research outcomes, in line with [17] as well as participant feedback on whether they felt they had benefitted from treatment overall and in what respect. However, further research is urgently needed to validate such thresholds and allow researchers to interpret their treatment outcomes accurately.

## Conclusion

This study is the largest trial involving people with MSA to date and the first to compare two different intervention procedures. It demonstrates that it is feasible to conduct a larger randomised controlled trial involving people with MSA-C as long as inclusion criteria are chosen in line with the length of follow up assessments to ensure adequate participant retention and changes in overall health status are factored into the outcome evaluation. Results also indicate that intensive intervention can be acceptable even to those at more advanced stages of their disease progression.

Our study adds to the growing evidence that speech intervention can be beneficial to people with MSA-C. Furthermore, the outcomes of the ClearSpeechTogether arm have demonstrated the potential to extend these benefits beyond the intervention period by introducing client-led group designs that support self-management and psychosocial wellbeing.

The results suggest that ClearSpeechTogether could be a feasible approach to provide acceptable intensive input without increasing clinician workload, as called for by Sackley et al. [17]. Furthermore, there is early indication that benefits might extend beyond those with ataxic symptoms, for example, for people with hypokinetic symptoms.

More definitive evidence in the form of properly powered RCTs is now needed to confirm the above assumptions and provide the evidence that allows health care providers to make decisions on the most appropriate intervention approach.

## Supplementary Information

Below is the link to the electronic supplementary material.

Supplementary Material 1

## Data Availability

All data underpinning this publication are openly available from the University of Strathclyde KnowledgeBase at 10.15129/9165803b-4a79-42f6-8aaf-915fcab2a7fe. Access to soundfiles can be granted upon request to the first author for participants who consented to this.
