# Supplementary material for: Speech Treatment for People with Cerebellar Multiple System Atrophy (MSA-C): A Pilot Randomised Controlled Trial of Two Approaches
Source: Cerebellum. 2025 Aug 14;24(5):142. doi: 10.1007/s12311-025-01895-y (PMC12354574; doi:10.1007/s12311-025-01895-y)
Supplement: Supplementary file 1 — Supplementary Material 1 [file 12311_2025_1895_MOESM1_ESM.docx]

**Supplemental materials**

Table S1a: Participant information relating to onset of symptoms and diagnosis

| ID | Arm | Gender | Age | Years since diagnosis | Years since motor symptom onset | Years since dysarthria onset | Difference between motor onset and diagnosis | Difference between motor and speech onset |
| --- | --- | --- | --- | --- | --- | --- | --- | --- |
| 1 | ST | female | 67 | 1 | 3 | 1 | 2 | 2 |
| 2 | ST | female | 58 | 1 | 4 | 2 | 3 | 2 |
| 3 | CST | female | 75 | 2 | 6 | 5 | 4 | 1 |
| 4 | CST | male | 49 | 3 | 6 | 6 | 3 | 0 |
| 5 | ST | female | 59 | 4 | 7 | 1 | 3 | 6 |
| 7 | CST | male | 58 | 3 | 5 | 5 | 2 | 0 |
| 8 | CST | male | 57 | 1 | 2 | 2 | 1 | 0 |
| 9 | CST | female | 62 | 2 | 9 | 3 | 7 | 6 |
| 10 | ST | female | 61 | 5 | 8 | 8 | 3 | 0 |
| 11 | ST | female | 70 | 2 | 5 | 1 | 3 | 4 |
| 12 | CST | female | 67 | 3 | 5 | 4 | 2 | 1 |
| 13 | ST | female | 64 | 0 | 1 | 0.5 | 1 | 0.5 |
| 14 | ST | male | 79 | 0 | 3 | 0.5 | 3 | 2.5 |
| 15 | CST | female | 73 | 2 | 5 | 0.5 | 3 | 4.5 |
| 16 | CST | male | 67 | 0 | 8 | 8 | 8 | 0 |
| 17 | CST | female | 64 | 0 | 4 | 4 | 4 | 0 |
| 19 | ST | female | 69 | 0 | 3 | 2 | 3 | 1 |
| 21 | CST | female | 65 | 0 | 5 | 5 | 5 | 0 |
| 24 | ST | male | 69 | 3 | 4 | 4 | 1 | 0 |
| CST | Mean (SD) | 6 F, 4 M | 63.7 (7.7) | 1.6 (1.3) | 5.5 (1.9) | 4.3 (2.1) | 3.9 (2.2) | 1.3 (2.2) |
| ST | Mean (SD) | 7 F, 2 M | 66.2 (6.6) | 1.8 (1.8) | 4.2 (2.2) | 2.2 (2.5) | 2.4 (0.9) | 2.0 (2.0) |

Abbreviations: CST: ClearSpeechTogether, ST: standard SLT, F: female, M: male, SD: standard deviation, SARA: Scale for the Assessment and Rating of Ataxia

Table S1b: Participant information relating to the main speech features and SLT input

| ID | Arm | DDK rate | MPT | Voice quality | Articulation rate | Irregular rhythm | Speech severity (SARA speech score) | Previous SLT input for speech | Number of sessions |
| --- | --- | --- | --- | --- | --- | --- | --- | --- | --- |
| 1 | ST | WNL | Sev | WNL | WNL | Y | 2.3 | Y | 1 |
| 2 | ST | Mod | Sev | Mild | Mod | Y | 2.5 | Y | 2 |
| 3 | CST | Mild | Mod | Sev | Mod | Y | 3.8 | Y | 3 |
| 4 | CST | Mild | Mod | Mod | Mod | Y | 3.0 | Y | 3 |
| 5 | ST | Mod | Sev | Mod | Mod | Y | 2.3 | N |  |
| 7 | CST | Mod | Sev | Sev | Mod | Y | 3.0 | Y | 7 |
| 8 | CST | WNL | Mild | Mod | Mod | Y | 2.5 | Y | 6 |
| 9 | CST | Mild | WNL | Mild | Mild | Y | 1.0 | Y | 2 |
| 10 | ST | Mod | Sev | Mild | Mod | Y | 3.8 | Y | 1 |
| 11 | ST | Mild | Mild | Mild | Mild | Y | 2.3 | Y | 8 |
| 12 | CST | Mod | Sev | Mild | Mod | Y | 2.5 | Y | 4 |
| 13 | ST | Mild | WNL | Mod | Mild | Y | 0.0 | N |  |
| 14 | ST | Mild | Mild | Mod | WNL | Y | 0.5 | N |  |
| 15 | CST | Mod | Sev | Mild | Mod | Y | 3.0 | Y | 2 |
| 16 | CST | Mod | Sev | WNL | Mild | Y | 3.8 | Y | 5 |
| 17 | CST | Mild | Sev | Mod | Mild | Y | 1.3 | Y | 6 |
| 19 | ST | Mild | Mod | Sev | Mild | Y | 1.8 | Y | 4 |
| 21 | CST | Mod | Sev | Mild | Mild | Y | 3.3 | Y | 1 |
| 24 | ST | Sev | Sev | Mild | Mod | Y | 4.8 | N |  |

Abbreviations: ID: participant identifier, CST: ClearSpeechTogether, ST: standard SLT, F: female, M: male, SD: standard deviation, SARA: Scale for the Assessment and Rating of Ataxia, SLT: speech and language therapy, Mod: moderate, Sev: severe, WNL: within normal limits

Speech features scoring guide:

DDK (pata repetitions): Sev: 0-6 repetitions per 10 secs (reps), Mod: 7-13 reps, Mild: 14-20 reps, WNL : 21+ reps

MPT (prolonged /ah/): Sev: 0-5 sec, Mod: 6-10 sec, Mild: 10-14 sec, WNL: 15 + sec

Voice quality (**G**RBAS): Sev: 3, Mod: 2, Mild: 1, WNL: 0

Articulation Rate (reading): Sev: below 2 syll/sec, Mod: 2-3 syll/sec. Mod; Mild 3– 4 syll/sec, WNL: 4+ syll/sec

Supplemental Table S2a: Scores for individual GRBAS voice analysis categories (mean, SD):

| Task | GRBAS |  | CST | | | | ST | | | |
| --- | --- | --- | --- | --- | --- | --- | --- | --- | --- | --- |
|  |  |  | A1 | A2 | A3 | A4 | A1 | A2 | A3 | A4 |
| Sustained vowel | Grade | mean | 1.77 | 1.47 | 1.33 | 1.48 | 1.44 | 1.70 | 1.56 | 1.44 |
|  |  | sd | 0.90 | 0.68 | 0.71 | 0.75 | 0.75 | 0.91 | 0.89 | 0.80 |
|  | Roughness | mean | 1.33 | 1.17 | 1.13 | 1.11 | 1.04 | 1.33 | 1.19 | 1.22 |
|  |  | sd | 0.96 | 0.83 | 0.63 | 0.85 | 0.85 | 0.92 | 1.00 | 0.97 |
|  | Breathiness | mean | 0.53 | 0.43 | 0.60 | 0.44 | 0.07 | 0.19 | 0.19 | 0.15 |
|  |  | sd | 0.82 | 0.50 | 0.77 | 0.64 | 0.27 | 0.56 | 0.40 | 0.36 |
|  | Asthenia | mean | 0.90 | 0.93 | 0.73 | 0.44 | 0.56 | 0.78 | 0.48 | 0.52 |
|  |  | sd | 0.92 | 0.91 | 0.64 | 0.58 | 0.64 | 0.75 | 0.58 | 0.58 |
|  | Strain | mean | 0.70 | 0.63 | 0.47 | 0.41 | 0.89 | 0.93 | 0.74 | 0.85 |
|  |  | sd | 0.92 | 0.72 | 0.51 | 0.64 | 0.85 | 0.87 | 0.71 | 0.72 |
| Free speech | Grade | mean | 2.03 | 1.97 | 1.80 | 2.22 | 1.63 | 1.59 | 1.59 | 1.78 |
|  |  | sd | 0.76 | 0.72 | 0.71 | 0.80 | 0.79 | 0.80 | 0.64 | 0.75 |
|  | Roughness | mean | 1.47 | 1.57 | 1.50 | 1.74 | 1.30 | 1.07 | 1.30 | 1.30 |
|  |  | sd | 0.94 | 0.94 | 0.86 | 0.98 | 0.54 | 0.55 | 0.82 | 0.95 |
|  | Breathiness | mean | 0.83 | 0.80 | 0.97 | 1.00 | 0.48 | 0.48 | 0.41 | 0.78 |
|  |  | sd | 0.87 | 0.81 | 0.81 | 1.11 | 0.89 | 0.85 | 0.64 | 0.89 |
|  | Asthenia | mean | 1.20 | 1.20 | 0.97 | 1.41 | 0.89 | 0.81 | 0.78 | 1.26 |
|  |  | sd | 1.06 | 1.06 | 0.93 | 1.22 | 0.85 | 0.79 | 0.58 | 1.02 |
|  | Strain | mean | 1.17 | 1.23 | 0.83 | 1.52 | 0.89 | 1.00 | 0.78 | 0.78 |
|  |  | sd | 1.02 | 0.94 | 0.95 | 1.12 | 0.85 | 0.88 | 0.58 | 1.05 |

Abbreviations: CST: ClearSpeechTogether, ST: standard SLT, SD: standard deviation, A: assessment

Supplemental Table S2b: Friedman test results for pre-/post-therapy comparisons of GRBAS voice characteristics

|  | Sustained vowel | | Free speech | |
| --- | --- | --- | --- | --- |
|  | CST | ST | CST | ST |
|  | p | p | p | p |
| Grade | 0.188 | 0.902 | 0.161 | 0.623 |
| Roughness | 0.640 | 0.360 | 0.497 | 0.365 |
| Asthenia | 0.819 | 0.477 | 0.156 | 0.560 |
| Breathiness | 0.984 | 0.300 | 0.407 | 0.364 |
| Strain | 0.622 | 0.705 | 0.055 | 0.812 |

Abbreviations: CST: ClearSpeechTogether, ST: standard SLT, p: p-value

Supplemental Table S3: Participant reported speech outcomes (percentage of participants per group):

| **Outcome** | **CST** | **ST** |
| --- | --- | --- |
| Louder/stronger voice | 55 | 44 |
| Better rate management | 36 | 22 |
| Improved breath support or management | 64 | 56 |
| Increased intelligibility / clearer speech | 27 | 22 |
| Better pitch modulation | 18 | 0 |
| Improved voice quality | 18 | 22 |

Abbreviations: CST: ClearSpeechTogether, ST: standard SLT
